# The medical simulation blog: A pilot project in Italy

**DOI:** 10.1080/10872981.2021.1920089

**Published:** 2021-04-25

**Authors:** Francesco Dojmi Di Delupis, Paolo Pisanelli, Nicola Di Daniele

**Affiliations:** a Department of Systems Medicine, University of Tor Vergata, Rome, Italy; b Department of Emergency Medicine, Misericordia Hospital, Grosseto, Italy

**Keywords:** Simulation, blog, social media, debriefing, faculty

## Abstract

**Introduction**: In Italy, medical simulation is undergoing a phase of intense diffusion, establishing a more decisive and uniform role in medical education. Educators receive many opportunities to train in simulation education, but these provide little room for personal growth and collaboration. This could have a negative impact on education quality and the standardization of processes. Thus, we found a gap in new information technology use, specifically in the informal diffusion of medical simulation content knowledge. Using a blog platform, we identified a space in which people can disseminate information, share their experiences, criticisms, and perspectives.

**Approach**: From March 2016 to November 2019, we implemented a novel pilot project, creating the first Italian blog on simulations, dedicated to simulation educators. It contained the following main sections: communication, debriefing, simulation experiences, instructions for use, journal club, and psychology.

**Findings**: Multidisciplinary personnel contributed to the blog’s content. With over 70 posts, the blog accumulated 25,615 pageviews and 9,056 sessions, without promotional, monetary support or diffusion efforts. The average visitor session was 2.17 minutes long and the average pages viewed in a session was 2.83. Additionally, 30.5% of the users were returning visitors and 58.67% found the website through Google.

**Insights**: Despite the blog’s niche subject, the results were encouraging. The materials were not only meant for personal viewing, but also as a source for announcing public events (meetings and workshops). The project provided educators with an easy tool for continuous education. We believe that it enabled and organized the informal sharing of educational simulation content. As such, it also offered significant insights into formal program consolidation and the standardization of simulation instruction, while we wait for further local scientific literature production. For future developments, we believe that collaborations with other stakeholders, scientific societies, and ethical sponsorship could foster this project’s continuation.

## Introduction

Medical simulation is a ‘social’ teaching method that recreates simulated or parallel realities, in complete safety, without harming the patient or endangering the operators. Simulation creates the possibility of studying and correcting the behavior of an individual, a team, and the system. In other words, simulation is a 360-degree tool for studying reality, based on its needs, and addressing specific learning and research objectives. It can highlight and identify some of the latent errors present in healthcare environments[1], correcting their root causes and increasing overall performance.

Simulations initially emerged in Italy for anesthesia and resuscitation[2], emergency medicine[3], and surgery[4] purposes. Presently, the approach is rapidly spreading to other medical disciplines in which system complexities and human factors could be the root of errors.

Universities, hospitals, paramedics institutions established countless simulation educational programs. The development of these programs quickly revealed the necessity for educators to conduct more structured training sessions. This resulted in the establishment of numerous simulation courses for educators, offered throughout the country by different providers [5–13]. These courses differed from one another in terms of educational objectives, methodology, duration, and faculty development.

Due to the pressure of these multidisciplinary initiatives, public and private institutions acknowledged the need for documentation and rules that regulate the branches of activity. Established in 2010, the Italian Society for Simulation in Medicine (SIMMED)[14], affiliated with the Society for Simulation in Healthcare (SSH), began a standardization process by promoting and encouraging training events, and disseminating information about cultural and scientific initiatives and innovation. Despite the fact that SIMMED published a list of minimum curricular requirements for simulation educators in their position paper[15]; because of the numerous training programs and the continuous introduction of new trainers, organizational and content knowledge gaps need to be addressed before we can focus on standardized training for simulation educators.

Due to the novelty of simulation and lack of practical experience, some educators will be overwhelmed by formal training courses. In contrast to other teaching methods, effective simulations require an awareness of the significance of the debriefing sessions. More specifically, beyond learning the technical aspects of simulation such as, mastering the mechanical operations of advanced simulation manikins; effective simulation educators require a shift in mindset from teacher as dispenser of information (sage on the stage) to teacher as facilitator (guide on the side)[16]. Simulation and debriefing require extensive advance preparation time and a high degree of self-confidence and awareness. Occasionally, health personnel who are short on time, training or experience are chosen for roles in simulation education. In these situations, there needs to be a support system of faculty development or appropriate mentoring[17]. Without support from mentors or a learning community of experts, these simulation educators will fail to effectively implement simulation programs.

If educators are not supported by mentors and a community of experts, they could lose some of the knowledge they acquired during their training, which could decrease simulation programs’ effectiveness.

As it is a relatively young subject in Italy, we found it necessary to establish a platform where people could update, compare, express, and share simulation ideas in a free, flexible, and informal way to stay up to date with international experiences and avoid the risk of content knowledge fragmentation and dispersion. Thus, this platform should avoid being bogged down by lengthy formal content, allowing a more rapid diffusion of simulation educational contents in our country, while we wait for more traditional scientific literature publications.

In Italy, aside from the scientific literature, there are no resources for disseminating simulation educational content like some of the forms of social media available to educators in other countries [18,19].

Also, scientific literature and programmatic choices do not imply an inclination to include simulation information within social platforms or online and in Italy, web-based information with medical simulation is still rather scarce. SIMMED posts exclusive materials on its website, such as presentations and the society’s conference proceedings, some commercial websites advertising simulation equipment and devices include sponsored educational content resources[20]. Numerous websites belonging to public or private medical simulation centers principally advertise their activities and their training courses. Furthermore, when we entered the words ‘blog,’ ‘web log,’ or ‘blogging’ on PubMed, we found no literature referring to experiences in medical simulation in Italy. Some literature abroad supports the use of social media and blogs as professional tools to increase the dissemination of scientific subjects in real time[21], providing a versatile and flexible economic platform for e-learning[22]. Blogs, are the oldest form of social media[23] delivering open access to information[24] and are widely used in different areas of medical education as emergency medicine for professional development and education[25].

Blogs provide the opportunity to publish information in a variety of media[26] promoting professional development and knowledge exchange among health care providers [27,28]. In regard to medical simulation, clinicians are increasingly using social media for professional development and education, resulting in a high number of online resources in medical simulation as those presenting simulation scenarios [29–31]. The studies showed that simulation educators should consider engaging on these platforms for their own benefit and to help spread simulation education around the world[32]. In this sense, blogs are easy-to-use tools that can enhance health professionals’ skills[33]. They could definitively serve as a simple and inexpensive way to exchange information, and reinforce theories and opinions regarding medical simulation, as is the case for other subjects in healthcare[34].

We hypothesized that an online tool (blog) could provide the mentoring and support needed by simulation educators in Italy and would eventually lead to active participation of health care educators and the promotion of simulation standards and training. Accordingly, the purpose of this paper is to describe the development of a novel independent blog of educational contents in medical simulation, www.simulazionemedica.com, dedicated to medical simulation educators. The blog aims to integrate and implement a virtual space where simulation operators or even enthusiasts can discuss, and provide experiences, opinions, perspectives, and constructive criticisms to maintain a non-sponsored and genuine standardized content.

## Approach

### Establishing the website

We posted the blog in March 2016. We designed a very simple web project that could be adapted and improved over time, focusing on content rather than effects. Technically speaking, we chose to build a blog using a simple template, without professional graphics or advanced options. A professional logo was the gift of an artist. The blog’s management, technical features, web hosting servers, editorial settings, content updating, and essential service maintenance (web domains, mail, and privacy policy) were entrusted to a volunteer web manager. Thus, the graphical interface has remained functional, yet very simple and quite lacking in advanced technicalities typical of corporate and profit-oriented websites.

### Voluntary participation

We decided to omit commercial sponsors to keep the information as independent as possible and create a space where people could share their experiences and cultural perspectives. Thus, the site’s financial management was completely independent and based on the founders’ voluntary contributions. We also chose to exclude paid tools that promote indexing on search engines. Furthermore, we deliberately did not perform web campaigns or site promotions, to determine if and how the blog could spread naturally across the user community.

Regarding the articles, we encouraged a practical, direct, and informal tone, pushing authors to put their personal experience into writing, while adding advice and examples. We preferred to use a multidisciplinary and multi-professional approach to encourage participation of simulationists from outside of health care such as aeronautics or psychology.

The blog’s main rule was to only publish articles that could teach others through personal experience. Contributors submitting to the blog were multidisciplinary educators involved in simulation education as doctors, nurses, paramedics, psychologists, airline and military pilots and make-up artists.

### The blog’s content

We divided the blog into six sections: 1) communication, 2) debriefing, 3) simulation experiences, 4) instructions for use, 5) journal club, and 6) simulation and psychology.

The communication section contained articles on all content related to the general topic of communication in healthcare.

In the debriefing section, considered the most important, we gave precedence to posts and articles related to the methodological aspects of debriefings. This section also served to propagate debriefing facilitators’ awareness of their important and crucial roles.

The simulation experience section had the greatest multi-and transdisciplinary openness, encompassing different ways of practicing simulation.

Instructions for use explained general simulation rules and procedures, while the journal club section offered critical readings of national and international literature on simulation and contained an area dedicated to readers’ comments.

Last, the simulation and psychology section’s function was to spread awareness of the importance of a safe educational environment, and to address simulation participants’ potential behavioral issues. In each section, contributors could post articles and multimedia content (audio or video). We also connected the blog’s content with social media platforms, such as LinkedIn and Facebook, for further dissemination of information.

### Content management

Two experts in simulation who had attended a training course in medical simulation at the Center for Medical Simulation (CMS)[35] in Boston carefully examined each post before publication, always with the consent and help of the authors, ensuring three quality indicators: credibility, content, and design. [36] For credibility, we reviewed for: 1) transparency about who was involved in the article’s creation, 2) qualifications of the creator to provide information on the topic, and 3) the author’s affiliation statement and independence from commercial sponsors or other conflicts of interest. For content we evaluated for the quality of the educational resources, including: 1) the professionalism of the article’s content, 2) its relevance for the blog’s audience, 3) the capacity to stimulate emotional engagement, and 4) the appropriate use of examples, scenarios, and cases to help readers understand the content. Finally, we assessed the article for quality of a design that promotes ease of reading and learning: 1) text formatting, font, layout, 2) choice of appropriate images to increase visual appeal, and 3) if audio was involved, the quality of the sound.

## Findings

We analyzed the volume of published content and collected traffic data through Google Analytics between March 2016 and November 2019. Google Analytics is a free web analytic service that provides detailed data on website visitor statistics. With this tool, we analyzed the total number and distribution of visitors, the geographical distribution of views, the behaviors of new visitors and returning visitors. We also examined the top viewed website categories and the most viewed articles.

Three years after its implementation, the blog had a total of 25,615 pageviews. The site only contained posts written in Italian, and therefore had an almost exclusive Italian viewership. However, there were a few visitors from outside of Italy including some from the USA (6.94%) and Brazil (2.37%). Within the date range, the total number of opened sessions was 9,056. New visitors accounted for 6,294 (69.50%) of these sessions and the remaining 2,762 (30.50%) were returning visitors. As for the percentage of geographical distribution of Italian visitors, central and northern Italy had the most and the top three cities being Rome (15.59%), Florence (12.73%) and Milan (12.35%). The average duration of a session was 2.17 minutes and the average number of pages viewed during a session was 2.83. First visitors averaged 1.39 minutes and 2.78 pages per session, while returning visitors’ data showed 3.44 minutes and 2.94 pages. Regarding the blog’s access methods, 58.67% of users entered through Google organic search, 25.49% through direct acquisition, 8.5% via social acquisition (Facebook, LinkedIn) and 7.3% through referral.

Multidisciplinary authors, such as doctors, psychologists, nurses, and pilots in military and civil aviation, have published over 70 posts, in Italian, in the blog’s various sections. The debriefing section contained 12 articles; average article length was 800 words. The blog also contained five audio materials. Top three consulted categories were: 1) simulation experiences, 2) debriefing and 3) instructions for use. The most viewed articles were: 1) fidelity, realism and simulation engagement 2) simulation as a didactic learning method and 3) the structured debriefing.

## Insights

Considering that the population of simulation instructors in Italy is quite small, and the fact that we did little website promotion, our blog encountered a high volume of users.

The geographical distribution of visitors is encouraging because it covers the whole Italian territory with a predominance for the main cities of the central and north of the country. This suggests that the blog has dispersed across a higher medical simulation population than expected. The average session length is also comforting, especially regarding returning visitors, who constitute a large percentage (30%) of our traffic. Additionally, professional orders, scientific societies, and other online journals in the health sectors have referenced some article links published on the site [37–40]. The blog material, with authors’ permission, has been used mainly for presentations during simulation courses, conferences and workshops[40]. This was encouraging evidence, that even in the absence of any promotional initiative or active signaling, the material’s propagation could take place spontaneously and naturally.

Although we are still in the pilot phase of publishing this web blog and have not yet collected evaluation feedback in a formal or scientific manner, we have received a fair amount of informal positive feedback. Educators have reported that the blog was a comprehensive source of practical information and we have been approached by simulation manufacturers for commercial collaboration. We are currently planning on adding content written in the English language, which we believe will increase the number of visitors and perhaps help connect our web blog to other web platforms across the world.

Debriefing articles were one of the top viewed sections of the blog. We believe that this is a positive outcome because debriefing is a vital component of simulations that should never be omitted[41]. Poorly facilitated debriefings may create adverse learning, produce negative perspectives and emotions, and lead to a degradation of the educator–learner relationship[42]. Different debriefing methods exist, but we believe that the most appropriate form is one that is conducted with good judgment, that allows healthcare workers and systems to evolve[43] and closes performance gaps[44]. In Italy, we think that this model could, at the same time, remove the punitive perception of errors, stimulate growth, and encourage an awareness of the facilitator’s role by helping the development and diffusion of reflective and critical thinking in healthcare environments. This could positively impact the clinical safety.

One limit of our pilot project was that professionals’ contributions are completely voluntary and uncompensated, including the time of the web manager, who currently contributes free time in the evenings and weekends to maintain the site. Should there be an increase in web blog traffic, it may be necessary in the future to either recruit more volunteer help or hire information technology personnel. To date, the deliberate choice to exclude corporate sponsorship was made to sustain an independent site, free of commercial interest. However, our project as at that tipping point, where we may be required to hold a fundraising campaign or adopt some other business model to sustain our efforts. In the next phase, our hope is to raise financial support but remain true to our mission.

The most motivated professionals have become passionate regular contributors, and have agreed to publish their bio-sketches and personal photos on a public blog profile. These motivated collaborators, including doctors, nurses, psychologists, military and civil pilots, make-up artists, etc., revealed the multidisciplinary nature of simulation through their contributions. This was the foundation for the pilot project’s creation and its initial success.

However, we have noticed a resistance of the blog’s visitors to post articles. We assume that this is because they use the blog more for knowledge content consultation than they do for social sharing. In the future, we hope to explore different ways for improving the user’s experience through the provision of needs assessment surveys, demographic forms, or other methods of interactive communication[45], in order to better assess the impact of our initiative over time.

## Conclusions

We believe that this pilot project was an important initiative in spreading simulation educational contents without using cutting-edge technologies and their related commercial policies.

Our goal for the future, after this pilot phase, is to expand the project with better stakeholder involvement and the implementation of an editorial staff. This would be essential for the blog’s future development and maintenance.
